# Comparative analysis of surface coatings for human neural co-cultures identifies fibronectin as a robust MEA substrate

**DOI:** 10.1038/s41598-026-67677-6

**Published:** 2026-08-20

**Authors:** Isabell Karnatz, Ida Keller Skousen, Benjamin Schmid, Christian Clausen, Bjørn Holst, Heiko Zimmermann, Jeanette Wihan, Julia C. Neubauer

**Affiliations:** 1 Fraunhofer Institute for Biomedical Engineering IBMT, 66280 Sulzbach, Germany; 2 Fraunhofer Project Center for Stem Cell Process Engineering, 97070 Wuerzburg, Germany; 3 Molecular and Cellular Biotechnology/Nanotechnology, Saarland University, 66123 Saarbruecken, Germany; 4 Bioneer A/S, Kogle Allé 2, Hørsholm, 2970 Denmark

**Keywords:** hiPSC-derived neurons, Astrocytes, Coating, Micro-electrode array, Polymers, Fibronectin, Biological techniques, Biotechnology, Cell biology, Neuroscience, Stem cells

## Abstract

Human induced pluripotent stem cell (hiPSC)-derived neurons offer a promising, physiologically relevant alternative to animal-based neurotoxicity models. However, the influence of the surface coating, a basic but important variable, has often been neglected. Here, the impact of commonly used surface coatings including polymers (polyethylenimine, poly-L-ornithine, poly-D-lysine, poly-L-lysine) as well as extracellular matrix proteins (laminin, fibronectin, Matrigel) on NGN2 neurons, primary human astrocytes, and neuron/astrocyte co-cultures was systematically investigated. Neurons cultured on polymer-only coatings exhibited a less mature neuronal network. Interestingly, neurons on polymers with laminin, laminin only, fibronectin and Matrigel exhibited no differences in protein and gene expression but displayed different electrophysiological profiles in co-culture with human primary astrocytes. The peak in number of network bursts was reached at different time points, with fibronectin and Matrigel as early as week 2. Co-cultures on fibronectin exhibited stable cell-electrode coupling and robust activity patterns up to week 6, despite being rarely used in MEA. In a proof-of-principle experiment, laminin, fibronectin and Matrigel were further evaluated in a donor-matched hiPSC-derived co-culture system, supporting the observed effects. This study highlights the importance of the coating selection for establishing neural cultures, ultimately improving the maturation and robustness of hiPSC-derived neuronal models for drug development.

**Supplementary Information:**

The online version contains supplementary material available at 10.1038/s41598-026-67677-6.

## Introduction

 Despite significant advances in neuroscience drug discovery, a large proportion of drug candidates targeting the central nervous system fail in clinical trials often due to insufficient efficacy or unexpected toxicity^1,2^. One important contributor to this failure is the limited predictive power of preclinical models, which often rely on rodent systems or immortalized cell lines that fail to accurately recapitulate human physiology^3^.

Human induced pluripotent stem cell (hiPSC)-derived neurons have emerged as a promising tool to address this gap, offering the potential to model human neurodevelopment and disease in vitro^4–7^. Their application in in vitro pharmacological testing, however, requires robust assay systems that allow for functional validation of neuronal network activity.

Micro-electrode array (MEA) technology has become an essential tool in this context, enabling label-free, and long-term monitoring of neuronal electrophysiology with quantitative insights into activity and network connectivity. Various efforts have been made to develop hiPSC-derived MEA-based in vitro platforms for compound screenings, focusing on optimizing high-level variables such as cellular composition, e.g. combining hiPSC-derived neurons with hiPSC-derived astrocytes^8^; medium formulation^9^; acceleration of differentiation timelines^10^; or the generation of distinct neuronal subtypes^11,12^. While these studies have resulted in a variety of protocols, they may be overlooking some of the key contributors to neuronal performance, and most importantly in assay development: robustness.

Neurons are highly mechanosensitive cells that respond to their microenvironment via integrin signaling and mechanosensitive ion channels, translating matrix cues into changes in gene expression, cytoskeletal dynamics, synaptic development, and ultimately neuronal electrical activity^13^. Consequently, basic culture parameters such as substrate coating can strongly influence functional MEA results and should not be neglected alongside more complex protocol variations.

Commonly used coatings in neuronal cell culture range from synthetic polymers - solely or in combination with the extracellular matrix (ECM) protein laminin (LN)^8,10,14–18^- over single protein coatings (LN^9^ or fibronectin (FN)^19^ to ECM protein mixtures (Matrigel, brain-specific decellularized matrices^20,21^). Synthetic polycationic polymers such as polyethyleneimine (PEI)^22^, poly-D-lysine (PDL)^23^, poly-L-lysine (PLL)^24^, or poly-L-ornithine (PLO)^25^ create a high-density positive charge to the otherwise negatively charged culture surface via electrostatic adsorption, which then binds the anionic glycocalyx of the cells promoting adhesion. When additional coating with proteins is performed, the charged layer adsorbs the protein, converting an inert dish into a bio-adhesive scaffold^26,27^. Additionally, ECM proteins provide binding sites for cellular integrins, supporting neurite outgrowth and long-term survival while mimicking the brain’s natural ECM^28^. The choice of cultivation coating has been shown to influence neurite network formation and the electrophysiological properties of neurons^22,29^. For example, Lam et al. compared a brain-derived ECM with the non-tissue specific ECM MaxGel and a non-ECM coating PDL, and reported an enhanced network activity on the ECM coatings^29^. One recent study by Yang et al. examined hiPSC-derived motor neurons on MEAs using coatings including PLO-LN, PLO-LN-FN, PLO-LN-FN-Matrigel, PLO-Matrigel, and PEI. Their findings highlighted that cultures on PEI showed uniform network distributions and reduced variability in electrical activity, suggesting that even small changes in coating composition can have functional consequences^22^.

Apart from these studies, the role of surface coatings in shaping the formation of neuronal networks and their electrophysiological activity has been investigated only to a limited extent.

Furthermore, most MEA-based studies use a co-culture model with astrocytes, since they promote synapse formation, regulate extracellular homeostasis, and support network stability^30,31^. Overall, a co-culture model mimics more accurately the in vivo environment. However, most studies overlook the critical role of astrocytes within their models, although there is evidence that astrocytes exhibit distinct responses to different coatings too^32,33^.

To address this gap, we conducted a systematic comparison of commonly used surface coatings (PEI, PLO, PDL, PLL, LN, FN, Matrigel) for the cultivation of hiPSC-derived neurons, human primary astrocytes and their co-cultivation. We focused on NGN2-induced neurons, a rapid and widely adopted protocol for producing excitatory neurons from hiPSCs^34–36^. By providing electrophysiological insights using MEA technology, we explore the most critical parameter – neuronal functionality, and thereby extend a recent study of Li et al. which compared several coatings (PDL, PLO, LN, Matrigel and combinations) for NGN2 neuronal cultures, primarily focused on morphological outcomes^18^. In addition, we evaluated the three best-performing coatings in a proof-of-principle co-culture model using hiPSC-derived astrocytes donor-matched to the NGN2 neurons.

With this study we aim to identify coating strategies that promote robust, consistent network maturation and reproducible electrophysiological activity, thereby improving the standardization and translational potential of MEA-based human neuronal assays for preclinical drug discovery and toxicology testing.

## Results

### hiPSC-derived neurons show distinct morphology and proliferation depending on the coating

First, we investigated the effect of different surface coatings on the maturation of hiPSC-derived neurons. Synthetic or semisynthetic positively charged polymers PEI, PLO, PDL and PLL, the ECM proteins LN and FN, and Matrigel.

**Fig. 1:**
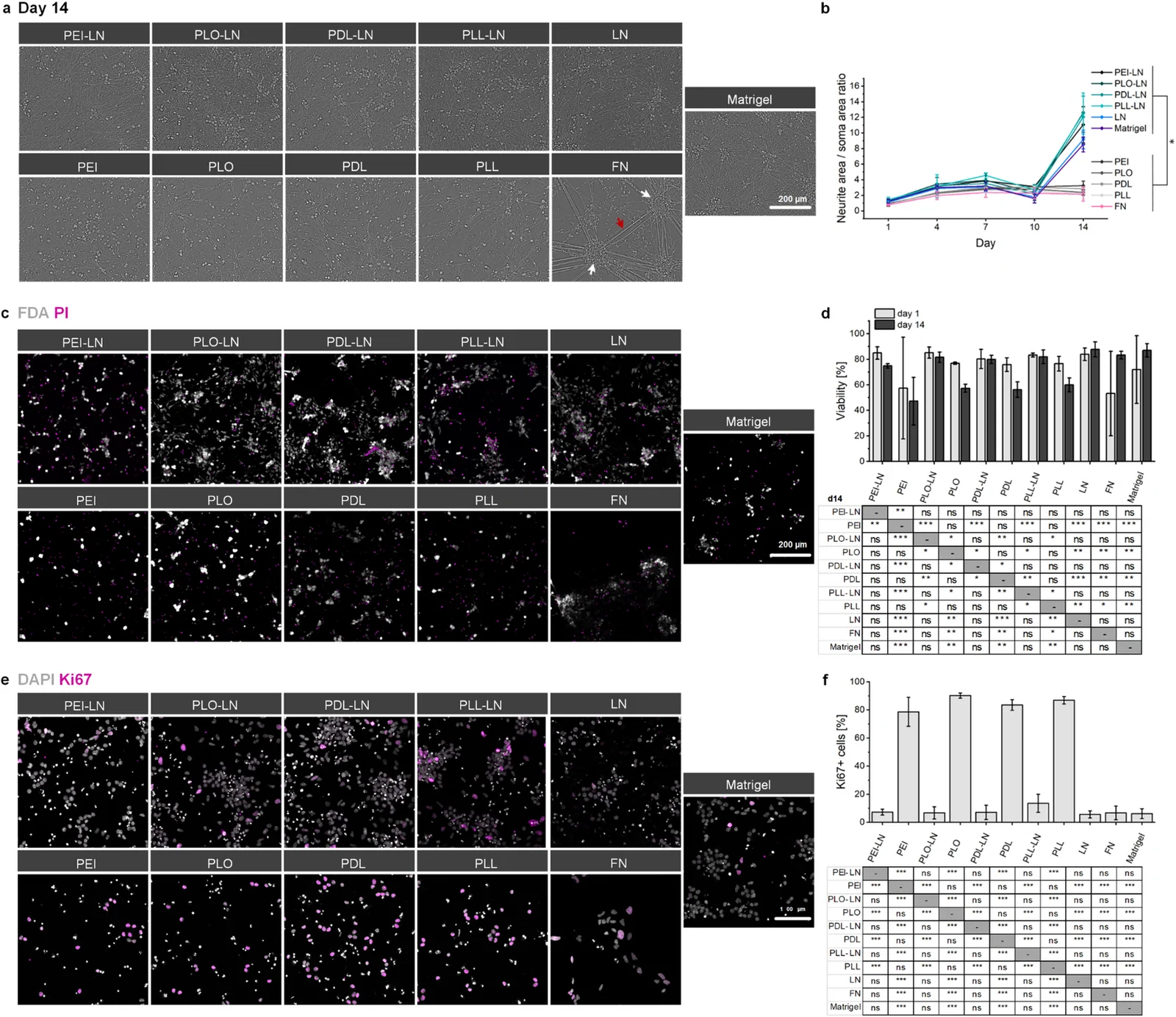
Effect of distinct coatings on neuronal morphology, viability, and proliferation over 14 days. (**a**) Bright field images of hiPSC-derived neurons on different coatings. Neurons cultivated on fibronectin (FN) showed enhanced aggregate formation (white arrow) and neurite bundles (red arrow). (**b**) Quantification of bright field images by analyzing the neurite area per soma area over a cultivation period of 14 days. Data is shown for day 1, day 4, day 7, day 10 and day 14 (N = 9; n = 3). (**c**) Representative images of FDA (live cells, gray) and PI (dead cells, magenta) staining of neurons on day 14. (**d**) Quantitative analysis of neuronal cell viability based on FDA/PI staining, presented as percentage of viable cells (N = 4, n = 3). (**e**) Representative immunocytochemistry images of neuronal cells on day 14 stained for DAPI and the proliferation marker Ki67. (**f**) Analysis of the percentage of Ki67 positive cells based on immunocytochemistry staining. Neurons cultured on PEI, PLO, PLL or PDL showed a significant higher proliferation compared to PEI-LN (N ≥ 5, n = 3). In d and f, a significance matrix is shown below the graph, indicating statistical comparisons. Data are presented as mean ± SD. Statistical analysis: One-way ANOVA followed by Tukey’s post hoc test. *p < 0.05; **p < 0.01; ***p < 0.001; ns, not significant. PEI, Polyethylenimine; PLO, Poly-L-Ornithine; PDL, Poly-D-Lysine; PLL, Poly-L-Lysine; LN, Laminin; FN, Fibronectin; FDA, fluorescein diacetate; PI, propidium iodid; DAPI, 4’,6-Diamidino-2-phenylinodole.

To analyze the morphology and neurite outgrowth of hiPSC-derived neurons, phase contrast images were captured on day 1, 4, 7, 10 and 14 after plating the cells (Fig. 1a). In general, neurons on all coatings developed a neurite network within 14 days. The network appeared tighter on PEI-LN, PLO-LN; PDL-LN; PLL-LN, LN and MTG compared to the remaining coatings. A striking difference in morphology was observed when cells were cultivated on FN. Here, the neurons formed large aggregates (Fig. 1a, white arrow) and thick neurite bundles (Fig. 1a, red arrow). The density of the neurite network was analyzed in more detail by image analysis and calculation of the neurite/soma area ratio (Fig. 1b). Until day 10 no significant differences in the ratio were detected between the coatings. On day 14, cells cultured on PEI-LN, PLO-LN; PDL-LN, PLL-LN, LN and Matrigel showed a significant higher neurite area compared to cells cultivated on PEI, PLO, PDL, PLL and FN, which corresponds to the observations made in the phase contrast images.

To exclude cytotoxic effects of the coatings, the viability was determined on day 1 and day 14 by FDA (live cells) and PI (dead cells) staining (Fig. 1c). Overall, cell viability ranged from 50% to 90% across all coatings. On day 1, the viability of neurons cultured on PEI (57.5 ± 39.8%) and FN (53.2 ± 33.1%) was the lowest. Interestingly, cell viability on PEI, PLO, PDL, and PLL decreased by ~ 20–25% until day 14 with values even below 60%. For cells cultivated on PLO-LN, PDL-LN and PLL-LN, the viability decreased only 0.5-5% over time and was still at around 80% on day 14. In contrast, cells on LN, Matrigel, and especially FN exhibited increased viability on day 14 with ~ 5% increase for LN, ~ 21% for Matrigel, and ~ 60% for FN compared to day 1. The cell viability for these coatings was around 85% on day 14, representing the highest value.

As hiPSCs differentiate into neurons, their proliferative potential decreases. To determine proliferation, neurons were stained for Ki-67 (Fig. 1e, f). Among all tested coatings, neurons on PEI, PLO, PDL and PLL exhibited the highest proportions of Ki-67 + cells, with values ranging from 80 to 90%. This trend was statistically significant to PEI-LN (*p* < 0.001). For PEI-LN, PLO-LN, PDL-LN, PLL-LN, LN, Matrigel and FN the proportion of Ki-67 + cells was below 15%, suggesting reduced proliferative activity.

In summary, PEI-LN, PLO-LN, PDL-LN, PLL-LN, LN, Matrigel and FN coatings supported neuronal viability and neurite extension while being associated with reduced proliferative activity.

### Astrocytes exhibit higher confluence and viability on LN, FN and Matrigel

 In vitro studies have shown that glia cells support functional synapse formation^37^ and that the co-cultivation with astrocytes leads to an increased network functionality of hiPSC-derived neurons^38^. Furthermore, astrocytes are mechanosensitive cells whose interaction with the surrounding microenvironment can alter astrocyte-mediated signal transduction, thereby influencing the homeostatic support of neuronal function^39^.

**Fig. 2:**
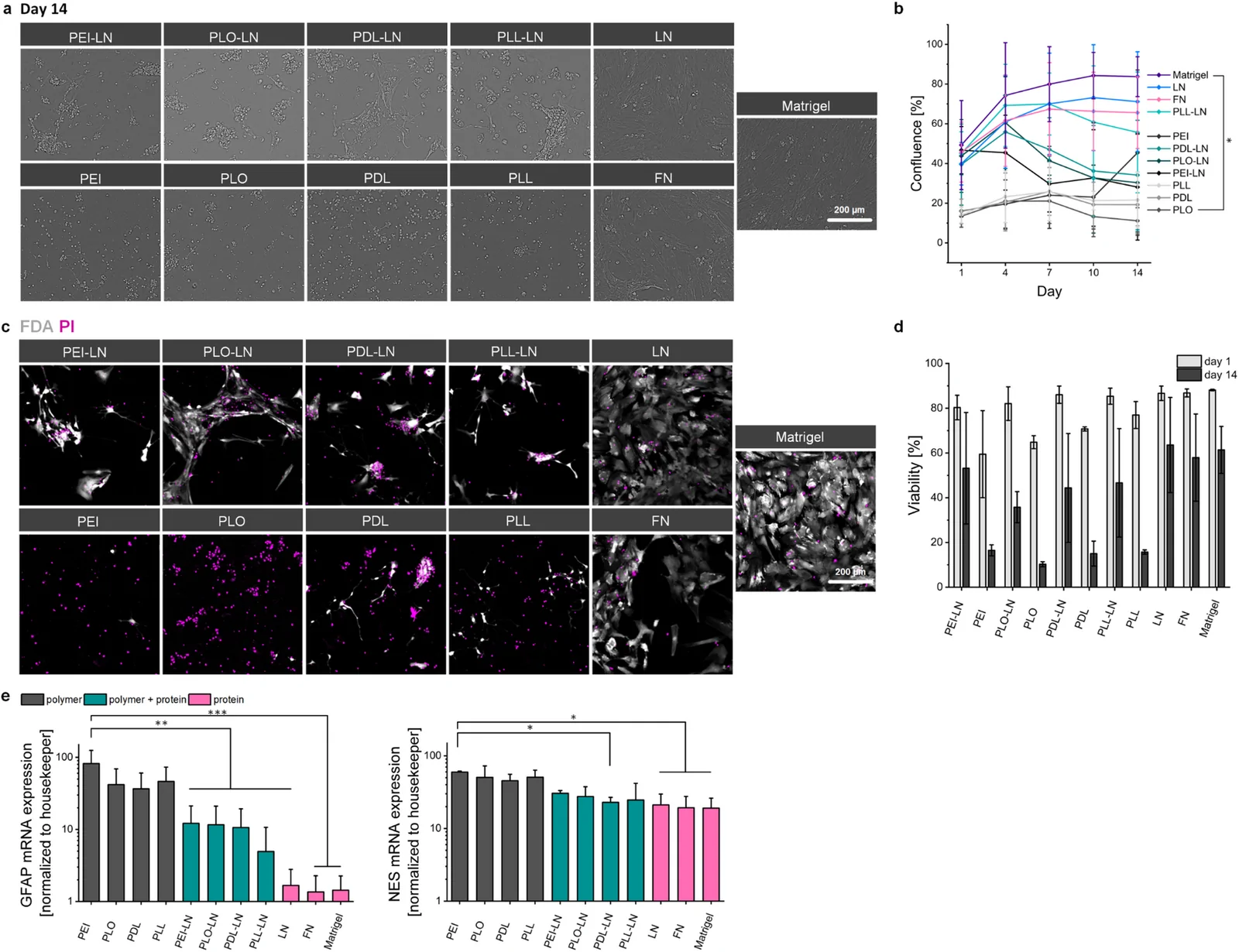
Cultivation of human primary astrocytes on distinct coatings over 14 days. (**a**) Representative bright field images. (**b**) Confluence of astrocytes over a cultivation period of 14 days. Cells cultivated on Matrigel, LN and FN showed the highest confluence by day 14. (**c**) Representative images of FDA (live cells, gray) and PI (dead cells, magenta) staining of astrocytes on day 14. (**d**) Quantitative analysis of astrocyte viability based on FDA/PI staining. (N = 5, n = 3) Data are presented as mean ± SD. (**e**) Gene expression analysis via RT-qPCR on day 14. Fold change of the gene expression was normalized to housekeeper genes (n = 3). Data are presented as mean + SD. Statistical analysis: One-way ANOVA followed by Tukey’s post hoc test. *p < 0.05; **p < 0.01; ***p < 0.001; PEI, Polyethylenimine; PLO, Poly-L-Ornithine; PDL, Poly-D-Lysine; PLL, Poly-L-Lysine; LN, Laminin; FN, Fibronectin; FDA, fluorescein diacetate; PI, propidium iodid; DAPI, 4’,6-Diamidino-2-phenylinodole.

We therefore explore the effects of the coatings on astrocytes alone through examining morphology, proliferation and viability. On LN, FN and Matrigel, the cells showed a spread-out and flat morphology (Fig. 2a). In contrast, on the remaining coatings, the astrocytes appear smaller, exhibit long processes and a more star-shaped morphology. On PEI, PLO, PDL, and PLL, the cells appear more isolated rather than forming confluent layers of cells as observed on the other coatings. This finding was also reflected in the confluence analysis (Fig. 2b). The confluence for PEI-LN, PLO-LN; PDL-LN; PLL-LN, PEI, PLO, PDL and PLL ranged between 10% and 50%, and for LN, FN and Matrigel between 70% and 90%. Comparing these results with the viability analysis reveals that coatings with higher cell confluence also exhibited increased viability (Fig. 2c, d). On day 1, the viability ranged from 75% to 90% for all coatings except of PEI, PLO and PDL, where the viability was lower already from the beginning, with values between 55 and 70%. Also, for these coatings and additionally PLL, a decrease in viability of ~ 80% could be observed from day 1 to 14.

Gene expression analyses were performed for GFAP, a marker associated with mature astrocytes, and Nestin, which is known to be re-expressed in reactive astrocytes^40,41^. This analysis was intended to provide an initial indication of whether astrocytes cultured on specific coating conditions exhibited features associated with a reactive phenotype. Astrocytes grown on polymer‑only coatings expressed higher levels of GFAP and slightly higher levels of NES than those cultured on any of the other coatings (Fig. 2e).

Due to the low viability of astrocytes on PEI, PLO, PDL and PLL, and the essential role for neuronal functions, we excluded these coatings from further analysis. This conclusion was supported by the observation that hiPSC-derived neurons on these coatings formed less developed neurite networks, exhibited a lower viability on day 14, and had a higher proportion of proliferating cells, indicating a lower degree of maturation.

### MEA recordings reveal distinct activity patterns of co-cultures depending on the selected coating

**Fig. 3:**
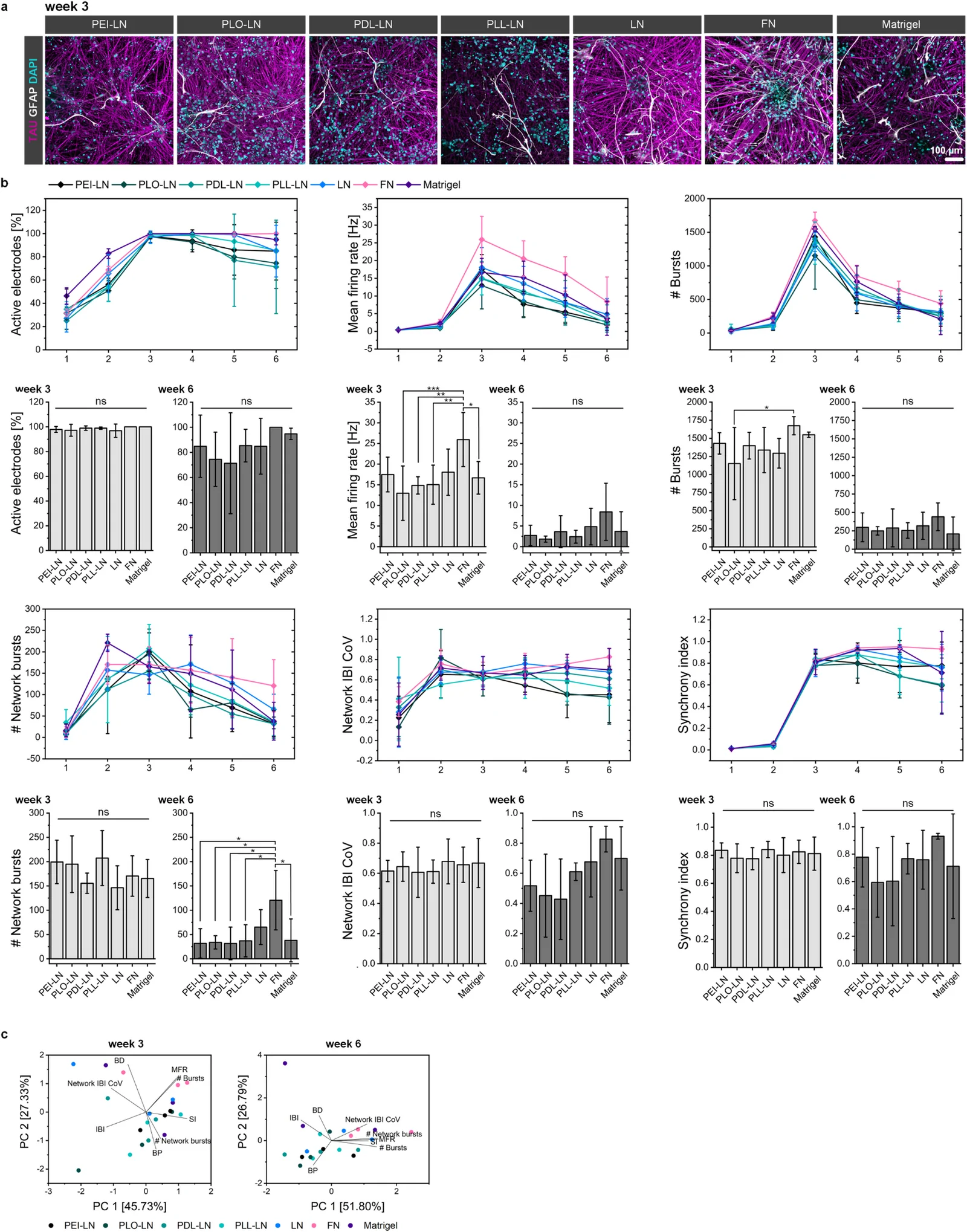
Electrophysiological maturation of hiPSC-derived neurons in co-cultures with astrocytes over six weeks. (**a**) Representative immunocytochemistry images of co-cultures of NGN2 neurons and human astrocytes on week three, stained for TAU, GFAP and DAPI. (**b**) Micro-electrode array (MEA) analysis of co-cultures over six weeks on different coatings for the parameters: active electrodes, mean firing rate (MFR, spikes per second), number of bursts, number of network bursts, network inter-burst interval (IBI) coefficient of variation (CoV) and synchrony index (SI). Data for week 3 and week 6 of each parameter is shown in the lower panels, and statistics were performed on these timepoints (*N* = 4 (PLO-LN: *N* = 3), *n* = 3, data is shown as mean ± SD). (**c**) Principal component analysis (PCA) on 8 MEA parameters for week 3 and week 6. Statistical analysis: One-way ANOVA followed by Tukey’s post hoc test. *p < 0.05; **p < 0.01; ***p < 0.001; ns, not significant; PEI, Polyethylenimine; PLO, Poly-L-Ornithine; PDL, Poly-D-Lysine; PLL, Poly-L-Lysine; LN, Laminin; FN, Fibronectin.

Since protein and gene expression analysis of NGN2 neurons on the different coatings revealed no significant differences (Supplementary Fig. 1), subsequent analyses focused on the evaluation of spontaneous electrical activity over time as a key functional readout of neuronal maturation. Therefore, NGN2 neurons were co-cultured with human primary astrocytes. To visualize the co-culture on different coatings, the cells were immunostained in week 3, as the highest overall electrical activity could be determined in this week (neurons TAU; astrocytes GFAP; Fig. 3a). While dense neurite networks were present on all coatings, the distribution and abundance of astrocytes varied depending on the coating, with FN showing a higher density.

The electrical activity of co-cultured NGN2 neurons was examined using the MEA technology (Fig. 3b). Representative spike raster plots are shown in Supplementary Fig. 2, while representative extracellular spike waveforms are provided in Supplementary Fig. 3. The percentage of active electrodes serves as a measure of the overall spread and homogeneity of neuronal activity in the MEA system. From week 3 on, the percentage of active electrodes is the highest in all conditions, ranging from 96.9% ± 5.4% for LN to even 100% ± 0% for FN and Matrigel. Until week 6 the percentage decreased to below 90% for PEI-LN (84.9% ± 24.8%), PLO-LN (74.5% ± 21.7%), PDL-LN (71.3% ± 40.2%), PLL-LN (85.4% ± 13.0%) and LN (84.9% ± 22.2%). For Matrigel it decreased to 94.8% ± 4.5%, only for FN the percentage of active electrodes was still at 100% ± 0% on week 6.

The overall neuronal activity is reflected in the MFR, which is calculated by the total number of spikes divided by the duration of the measurement. The MFR, reached its peak on week 3 and decreased until week 6 for all coatings. On week 3, the MFR was significantly higher on FN (25.9 Hz ± 6.5 Hz) compared to PLO-LN (13 Hz ± 6.6 Hz), PDL-LN (14.8 Hz ± 2.1 Hz), PLL-LN (15 Hz ± 4.7 Hz) and Matrigel (16.7 Hz ± 3.9 Hz). On all coatings the MFR decreased until week 6, while FN exhibited the lowest decrease (67.5%) compared to week 3 and PLO-LN the highest (86%).

Furthermore, the number of bursts and network bursts was analyzed. Bursts indicate the presence of structured, high-frequency spiking events, which are characteristic of developing neuronal networks and network bursts further highlight the degree of synchronized activity across multiple electrodes. On week 3, the highest number of bursts was detected on FN (1674.3 ± 125.7), which was significantly higher compared to PLO-LN with 1151.2 ± 498.7 bursts. On week 6, no significant differences between the coatings could be determined, but FN still exhibited the highest number of bursts (440.5 ± 188.8).

The coatings reached their peak in network burst number at different time points: Matrigel on week 2 (221 ± 20.2), FN on week 2 (170.3 ± 45.7) and week 3 (170.5 ± 41.7) with a similar number of network bursts, PEI-LN (199.4 ± 44.8), PLO-LN (194.8 ± 58.3), PDL-LN (112.8 ± 3.7) and PLL-LN (207.3 ± 56.6) on week 3, and LN on week 4 (170.8 ± 68.7). The decrease over time from the peak week to week 6 was lowest for FN with ~ 30%, followed by LN with ~ 60%. For the other coatings the decrease was ~ 80% until week 6. Additionally, cells cultivated on FN exhibited significantly more network bursts on week 6 compared to cells cultivated on PEI-LN, PLO-LN, PDL-LN, PLL-LN and Matrigel.

Additionally, the coefficient of variation for the inter-network burst interval (network IBI CoV) is displayed. It describes the regularity of network bursts to occur. Lower values represent a higher regularity whereas higher values indicate sporadic bursting. Week one is included for completeness; however, given the low number of network bursts and overall neuronal activity (MFR) the coefficient of variation at this time point is considered to have limited interpretative value. In general, the network IBI CoV is relatively stable from week 3 on for all conditions. It is notable, that PEI-LN, PLO-LN, PDL-LN, PLL-LN and LN showed the lowest network IBI CoV on week 5 or 6 indicating a more regular network pattern at longer cultivation duration. In contrast, cells cultivated on Matrigel exhibited the lowest CoV on week 4 (0.65 ± 0.19) and FN even earlier on week 3 (0.67 ± 0.12).

Ultimately, the synchrony index was analyzed. This is a unit-free parameter, which ranges from 0 (no synchrony) to 1 (perfect synchrony), and is calculated according to Paiva et al. in 2010^42^. The synchrony index increased strongly from week 2 to week 3 for all coatings. On week 3, the index was at ~ 0.8 for all coatings and increased by week 4 except for PEI-LN, here the index decreased from week 3 on. FN and Matrigel reached the highest synchrony index with 0.95 ± 0.02 and 0.93 ± 0.03 on week 5. Among all coatings, PEI-LN, PLL-LN, and FN exhibited the highest stability in synchrony index between week 4 and week 6, with only minor decreases of 2.9%, 12.6%, and 1.1%, respectively. Although no significant differences could be detected, it is noteworthy, that on week 6 FN still exhibited a high synchrony index with a lower standard deviation (0.93 ± 0.02) compared to all other coatings.

Given the large number of electrophysiological features extracted from MEA recordings, Principal Component Analysis (PCA) was used to reduce dimensionality and highlight overall trends and activity profiles that may not be apparent when considering individual parameters alone (Fig. 3c). On week 3, PC1 was primarily influenced by a negative loading of the IBI and network IBI CoV, along with positive contributions from the number of network bursts and synchrony index. This suggests that PC1 reflects network regularity and synchronization. In contrast, PC2 was driven by MFR, number of bursts, and burst duration, with a negative contribution from burst percentage (percentage of spikes within a burst). This component appears to capture overall activity levels, indicating a network that is active but less coordinated. The PCA biplot reveals that especially FN is distributed near the positive end of PC1 and PC2, indicating that the co-cultures on FN exhibited higher firing rates, increased burst activity, and greater synchrony, along with more structured bursts and rhythmic firing patterns. In contrast, PLO-LN, PDL-LN, and PLL-LN were primarily located towards the negative end of PC2, with PC1 values around 0, suggesting a low overall level of electrical activity and reduced synchrony of the co-cultures.

On week 6, PC1 became dominated by MFR, number of bursts, number of network bursts, and synchrony index, reflecting the overall neuronal activity. In contrast, PC2 was primarily shaped by burst duration, IBI, burst percentage and the network IBI CoV, indicating the temporal structure and regularity of the activity. Two cluster can be identified, one composed of the protein-only coatings (LN, FN, Matrigel) and one which contains the polymer coatings (PEI-LN, PLO-LN, PDL-LN, PLL-LN). Interestingly, the protein-only cluster revealed higher PC1 and PC2 values, suggesting that cells exhibited a higher excitability and more organized burst patterns.

### Temporal changes in the variability of co-culture network activity across coatings

**Fig. 4:**
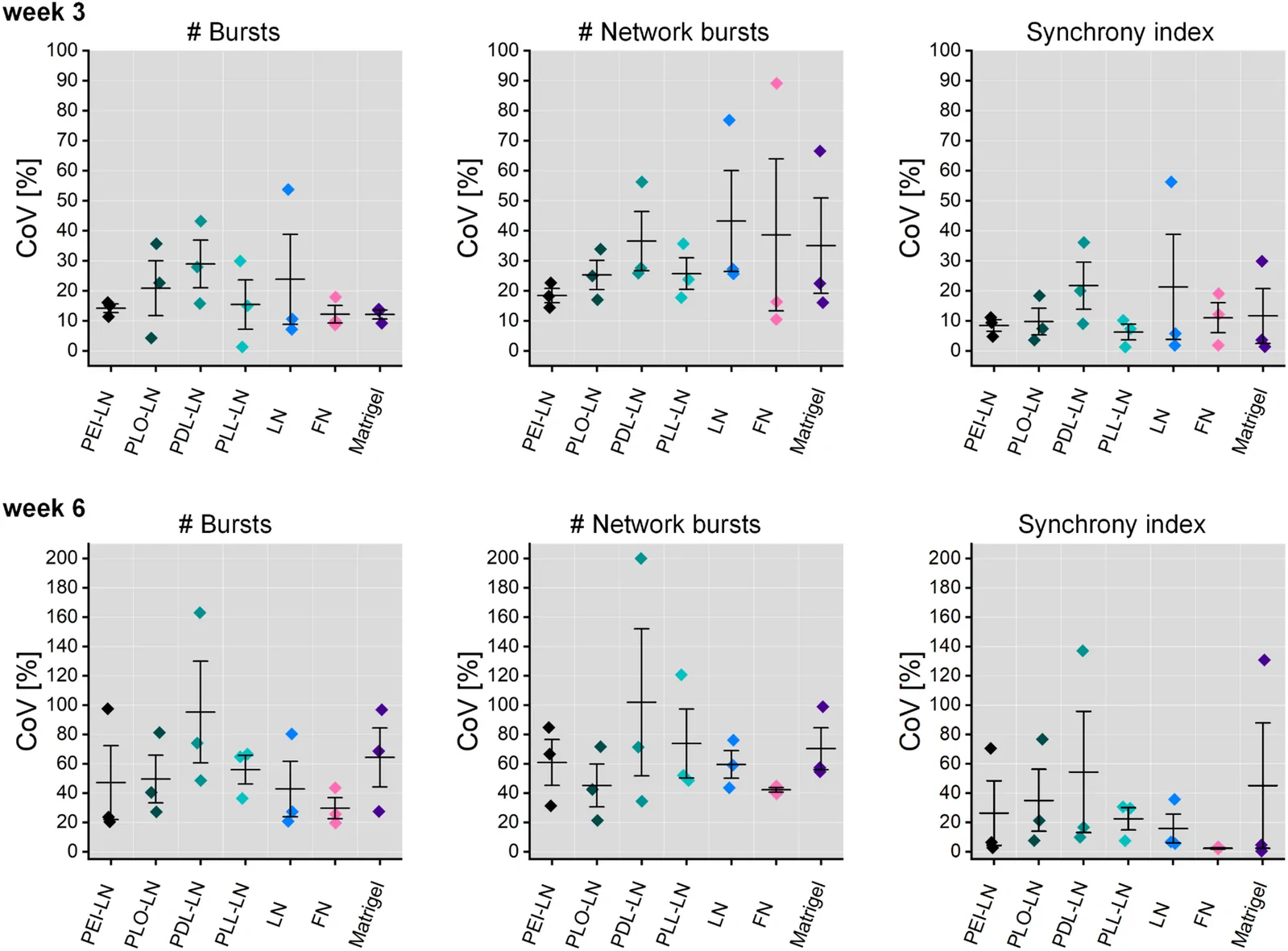
Inter-well and batch-to-batch variability of MEA analysis. Analysis was performed via calculation of the coefficient of variation (CoV) for the MEA parameters number of bursts, number of network bursts and synchrony index for week 3 and week 6. Each data point reflects the inter-well variability within a neuronal differentiation batch. Scatter plots indicate the overall batch-to-batch variability. (*N* = 4, *n* = 3 (neuronal differentiation batches), data is shown as mean ± SE with the three differentiation batches presented as routes). Statistical analysis: One-way ANOVA followed by Tukey’s post hoc test (*p* ≤ 0.05); results were not statistically significant. PEI, Polyethylenimine; PLO, Poly-L-Ornithine; PDL, Poly-D-Lysine; PLL, Poly-L-Lysine; LN, Laminin; FN, Fibronectin.

High reproducibility is essential for applications, such as compound screenings, thus, minimizing the inter-well and batch-to-batch variability plays a crucial role. Therefore, MEA data from three independent neuronal differentiation batches were analyzed at two different timepoints, revealing overall low batch-to-batch variability, although slightly increased variability was observed for some coatings at week 6 (Supplementary Fig. 4). For further validation, the coefficient of variation (CoV) was calculated (Fig. 4). A lower CoV represents reduced inter-well variability and thus greater consistency of the measured parameter. Each individual data point represents the inter-well variability within a differentiation batch, while the scatter plots with error bars illustrate the variability across independent differentiation batches. For the number of bursts on week 3, PEI-LN, FN and Matrigel exhibited the lowest CoV compared to the remaining coatings, indicating greater consistency of burst counts. Both inter-well variability and variability across neuronal batches were consistently low. In week 6, an increase in CoV was observed for PEI-LN, Matrigel, and FN. Only FN remained below 30%, while the CoV for other coatings ranged from 40% to 95%. The CoV for network bursts was highest on week 3 for PDL-LN (36.6% ± 17%), LN (43.3% ± 29.1%), FN (38.6% ± 43.8%) and Matrigel (35% ± 27.5). By week 6, the CoV values for LN, FN and Matrigel showed a slight increase while the batch variability became considerably smaller reflected by reduced standard deviations. Similarly, for the CoV of synchrony index the standard deviation increased across all coatings by week 6, except for LN and FN, where a reduction was evident.

### Validation of coating-dependent effects in donor-matched hiPSC co-cultures

**Fig. 5:**
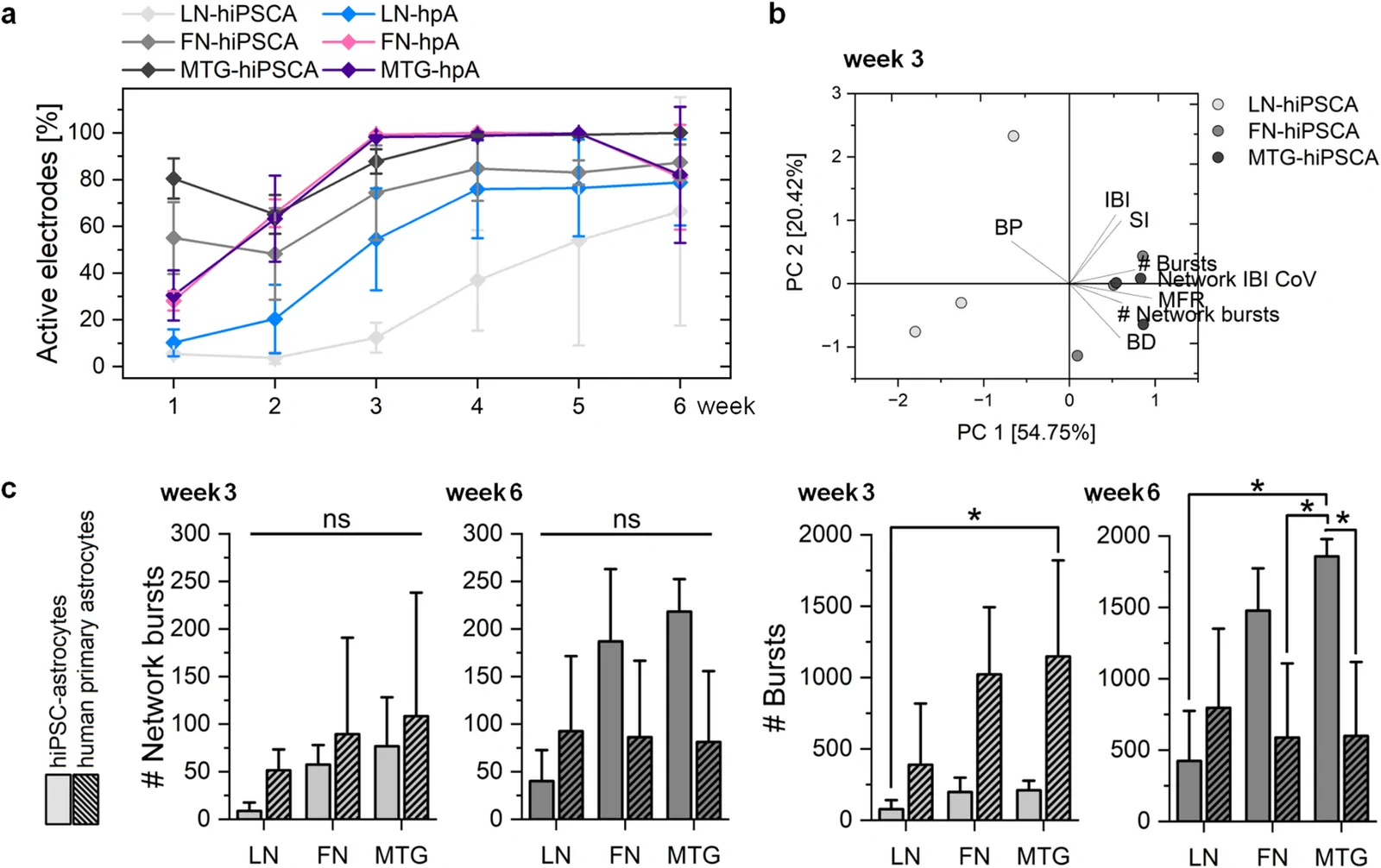
Micro-electrode array (MEA) analysis of hiPSC-derived neurons in co-cultures with hiPSC-derived astrocytes. (**a**) Percentage of active electrodes over a cultivation duration of six weeks. Cells were cultivated on LN, FN and MTG (*N* ≥ 5, *n* = 3, data is shown as mean ± SD). (**b**) Principal component analysis (PCA) on 8 MEA parameters for week 6 (MFR, mean firing rate; BD, burst duration; BP, burst percentage; IBI, inter-burst-interval; SI, synchrony index). (**c**) The mean firing rate, number of bursts and network bursts are displayed for week 6 (*N* ≥ 5, *n* = 3, data is shown as mean + SD). Statistical analysis: One-way ANOVA followed by Tukey’s post hoc test. *p < 0.05; ns, not significant; LN, Laminin; FN, Fibronectin; MTG, Matrigel.

Based on the initial MEA analysis with co-cultures using human primary astrocytes, the three most supportive coatings (LN, FN, Matrigel) were examined in an additional co-culture system with hiPSC-derived astrocytes matched to the neuronal donor line. This enabled the assessment of whether the coating-dependent effects are transferable to a fully donor-matched human system (Fig. 5).

The active electrode development over six weeks of cultivation showed coating-dependent differences, with Matrigel and FN supporting a more rapid increase in activity compared with LN (Fig. 5a). For cultures on all three coatings, the maximum percentage of active electrodes was reached at week 6, with only cells on Matrigel displaying a 100% active electrode rate (LN 66.4 ± 48.9; FN 87.3 ± 7.7; Matrigel 100 ± 0). Accordingly, PCA and selected MEA parameters are displayed for this timepoint. PCA demonstrated a clear separation of LN from FN and Matrigel (Fig. 5b), consistent with the trend observed for the percentage of active electrodes. PC1 was mainly driven by network activity parameters (e.g. MFR, number of bursts and network bursts), whereas PC2 reflected differences in burst structure (e.g. IBI, burst duration). Co-cultures on LN are clustered primarily along PC2, reflecting a highly active but less synchronized network. FN and Matrigel were characterized mainly by PC1, indicating a more synchronized network activity. The MFR and number of bursts were significantly higher in cultures on Matrigel compared to LN. For the number of network bursts, no significant differences between the coatings could be detected (Fig. 5c). However, the number of network bursts was ~ 5 times higher for cultures on FN (186.9 ± 76.1) and Matrigel (218.3 ± 34.1) compared to LN (40.2 ± 32.6). Overall, cultures on FN and Matrigel showed comparable results without significant differences.

## Discussion

The present study demonstrates that the surface coating choice can critically influence the functional network development of hiPSC-derived NGN2 neurons in co‑culture, and thereby supports and extends previous work^18,22,29^.

Overall, the combination of morphological assessments, viability measurements, neurite growth analyses and proliferation data differentiated the tested surface coatings into two functionally distinct groups. Neurons cultivated on polymer-only coatings showed lower values for neurite outgrowth and viability, and higher values for proliferation analysis, suggesting a less developed and immature differentiation state. Whereas neurons cultivated on polymers with LN, only LN, FN and Matrigel exhibited higher values for neurite outgrowth and viability, and lower values for proliferation analysis, indicating a more mature differentiation state. These findings are consistent with those of Li et al., who were able to distinguish between polymer- (PDL, PLO) and ECM coatings (LN, Matrigel) regarding neurite outgrowth^18^. Likewise, Sun et al. demonstrated that FN and Matrigel, among others, enhance neurite length and network assembly^43^. Our findings together with these studies suggest that ECM protein-based coatings accelerate neuronal network formation compared to polymer-only coatings likely by providing key biochemical cues of the brain microenvironment.

Furthermore, the viability of neurons on polymer-only coatings decreased strongly over the cultivation time. The cytotoxicity of polymer coatings has already been demonstrated^44^. In the present study, extensive washing steps were applied to minimize residual toxicity, however neuronal viability remained reduced. In comparison, the viability of neurons on LN, FN and Matrigel increased over cultivation time, with the highest increase observed for neurons cultivated on FN. In addition, the neuronal morphology differed strongly on FN coating showing somata aggregation and neurite bundle formation. These findings are consistent with the literature, were FN is reported to promote neuronal outgrowth and axonal survival^19,45^.

FN interacts with cells primarily via integrins and thereby activates integrin-mediated signaling pathways. Specifically, binding of FN to α5β1 integrins is known to trigger survival signaling, including phosphorylation of focal adhesion kinase (FAK) and upregulation of the anti-apoptotic protein Bcl-2, ultimately reducing apoptosis. This mechanism likely contributes to the improved neuronal survival observed in our study^46^. Time-resolved analysis of FN distribution in neuronal cultured as well as functional perturbation experiments, such as the use of integrin-blocking antibodies, to disrupt integrin signaling pathways would help clarify whether the observed effects are indeed mediated by integrin-FN interactions, and would provide deeper insight into how NGN2-induced neurons interact with FN and potentially restructure ECM in vitro^47,48^.

Similar to neurons, astrocytes also responded depending on the coating substrate, which is consistent with earlier work by Johnson and Miller, who showed that the culture substrate can influence, for example, the migratory ability of astrocytes, depending on the ECM protein present^49^. In the present study, astrocytes cultured on polymer-only coatings exhibited a slightly reactive phenotype. They grew isolated and formed long processes on polymers, which is in line with the hypertrophic morphology of reactive astrocytes typically associated with cellular responses to e.g. inflammation^50^. This observation was further supported by RT-qPCR analyses, which showed increased expression levels of GFAP and NES. Interestingly, while astrocyte reactivity is commonly induced by inflammatory cytokines in vitro^51^, our findings suggest that also the coating can promote reactivity.

The most important characteristic of mature neurons is their electrical activity, which can be used in pharmacological studies to evaluate the impact of active substances, making it one of the most significant parameters. To test whether the coatings affect the functional maturation of the neuronal network, MEA analyses were performed in co-cultures with astrocytes. Neuronal networks developed comparable levels of spontaneous electrical activity across all tested surface coatings. The percentage of active electrodes was the highest on week 3 for all coatings. FN sustained 100% electrode activity through week 6, implying robust cell adherence and a homogeneous monolayer. Given the importance of cell-electrode coupling for MEA recordings, the data suggest a strong coupling on FN. In line with this, Braun et al. reported a shorter distance between neural cells and FN (60 nm) compared to LN (159 nm), which supports the observed performance differences^52^.

Notable, the cells reached their highest number in network bursts at different time points. These differences imply that the optimal assay window for compound testing can depend on the chosen substrate, and furthermore, that the optimal assay window of a functional cell model can be shifted by changing the culture substrate. Researchers requiring an earlier readout may favor FN or Matrigel (week 2), whereas polymer coatings with LN yield more regular and synchronous networks at later stages.

Neurons cultured on LN and FN generated the highest numbers of network bursts at week 6, suggesting that they more effectively promote the development of a functional bursting network. Yet the number of network bursts alone cannot capture the full electrophysiological phenotype, therefore a comprehensive assessment of multiple MEA parameters is essential. When several parameters were integrated in a PCA, two distinct activity profiles among the tested surface coatings could be slightly distinguished: one formed predominantly by ECM protein coatings (LN, FN, Matrigel) and another composed largely of ECM-polymer combinations (PEI-LN, PLO-LN, PDL-LN, PLL-LN). These results emphasize that although co-cultures on LN, FN and Matrigel differ in single parameters, such as network bursts, the electrophysiological pattern is largely comparable. Additional replicates may further enhance the resolution of coating-specific clusters in PCA. Overall, PCA is a powerful tool for MEA data analysis, as has already been shown by Mossink et al.^16^. To further evaluate the functional connectivity, advanced analyses such as correlation-based functional connectivity matrices between electrodes can be used to quantify the strength of interactions within the network. Analyses of burst propagation patterns and the identification of “leader” electrodes that consistently initiate network bursts may reveal how activity spreads across the array and how this is influenced by different coatings^53^.

An additional readout for assessing neuronal maturation would be spike-shape analysis. Waveform properties can provide information beyond network-level activity measures and may reflect developmental changes at the single-neuron level^54^. However, due to the planar MEA system with limited spatial resolution used in this study, as well as the high cell seeding density required for robust network formation, the present work focused on neuronal network development rather than waveform properties at the single-electrode level. Future studies using higher-resolution recording platforms or complementary electrophysiological approaches may help to further resolve spike-waveform maturation in NGN2 neurons under different cultivation conditions.

To assess the reproducibility of neuronal electrical activity, the CoV for key MEA parameters, such as bursts, network bursts and synchrony index was shown. Interestingly, a decrease in variability from week 3 to week 6 for all three parameters was shown by FN and LN, as indicated by a substantial decrease in standard deviation. These findings suggest that ECM-only coatings tend to enhance the robustness of functional readouts in long-term neuronal culture. Further, the data emphasizes that the coating influences both functional maturation and the variability of neuronal activity across experiments, which is an important aspect for the standardization of in vitro models.

To further reduce inter-donor variability, donor-matched neuron-astrocyte co-cultures represent an important next step in hiPSC-based assay development. Therefore, the three most promising ECM coatings (LN, FN, and Matrigel) were tested in a proof-of-principle study in such a donor-matched co-culture system. MEA recordings revealed higher network activity on FN and Matrigel compared to LN, reflected by increased MFR and burst number. In addition, the PCA, loaded with eight different parameters, indicated a less synchronized network on LN compared to FN and Matrigel. Notably, FN and Matrigel did not differ significantly and exhibited comparable activity profiles, indicating that both provide similarly supportive environments for the formation of functional neuronal networks.

It is important to note that in all experiments of this study human FN was used, whereas LN and Matrigel are of murine origin, resulting in a not fully human cell model. While future studies should evaluate human recombinant brain-specific LN isoforms, these data already identify FN as a suitable substrate for hiPSC-derived co-culture models.

However, the physiological relevance of FN remains uncertain. In the healthy adult brain, FN is largely confined to the vascular basement membrane and its expression increases in glial scars following injury^55–57^. FN is also transiently present during cortical development, expressed by migrating cortical neurons before being downregulated as the cortical plate differentiates^58^. As NGN2-induced neurons represent post-mitotic but still immature cortical neurons, an FN-rich substrate may reflect aspects of the developmental environment rather than the adult CNS parenchyma. Although FN is associated with glial scars, astrocytes cultured on FN did not exhibit reactivity-associated features. Instead, they displayed a flat, spread morphology and lower GFAP and NES expression than astrocytes grown on polymer-only coatings, further supporting a developmental rather than injury-related interpretation. FN is therefore proposed here as a suitable substrate for improving the robustness of MEA assays, rather than a material intended to recapitulate the composition of healthy adult CNS tissue.

As LN is widely used in neuronal cell culture systems, placing the present observations in the broader context requires careful consideration. Much of the available evidence, derives from neural stem and progenitor cells, in which LN has been shown to promote expansion, migration, and differentiation^59,60^. Ma et al. reported that LN and LN-rich Matrigel outperformed FN for neural progenitor generation and neurite outgrowth from human embryonic stem cells^60^, and Flanagan et al. found LN superior to PLO, FN and Matrigel for the migration, expansion and differentiation of human neural stem and precursor cells^59^. These studies are only partially transferable to the present model, since NGN2 induction generates post-mitotic excitatory neurons directly. Accordingly, fewer than 15% of cells on the ECM-containing coatings were Ki-67 positive. Moreover, the mentioned studies show that substrate preference depends on neuronal subtype and origin. In adult mouse cortical and hippocampal neurons for example, FN supported greater neurite outgrowth than LN^19^. An overview of the studies most relevant to the present work, including neuronal subtype, origin, coatings tested and outcome measures, is provided in Supplementary Table 1. This table includes only studies that explicitly compared different coatings, and it illustrates that the “best” coating varies considerably across species and neuronal subtypes. Moreover, in most of these studies the preferred coating was defined primarily by neurite outgrowth and other morphological or survival metrics, whereas electrophysiological measures were rarely assessed^18,43,60^. Yet, for functional applications such as compound testing, electrophysiological activity is one of the most relevant readouts. Yang et al. already noted that coating choice should depend on the experimental goal, with PLO-Matrigel being more suitable for rapid maturation and PEI for uniformity in MEA analysis^22^. Sun et al. explicitly concluded that no universal best coating exists that fits all purposes^43^. To date, no study has systematically assessed how different coatings influence the electrophysiological network activity of NGN2-induced human cortical neurons, although these cells have become a standard tool in the field^34^. In this context, the present data do not claim FN as a universally optimal coating, but identify it as particularly well suited to MEA-based measurements in NGN2 neuron-astrocyte co-cultures, without implying that it is universally superior to LN or polymer-based composites.

Certain limitations of this study should be considered when interpreting the findings. First, the present findings were conducted using NGN2-induced cortical neurons, a rapid transcription factor-driven differentiation process that bypasses intermediate stages of precursor cells and therefore does not fully reflect all aspects of physiological neurodevelopment^34^. Accordingly, optimal culture conditions, including substrate coating and maturation time, may differ depending on the neuronal differentiation protocol (e.g. dual-SMAD induction^61,62^. Here, an overall activity decrease after approximately 3–4 weeks until week 6 was observed. Studies that used dual-SMAD induction protocols instead performed MEA measurements up to 36 weeks without major decline in activity^63^. In contrast, studies employing NGN2 induction typically report activity windows similar to those described here: Mehrkanoon et al. observed a peak in total spikes around day 28 followed by a decrease until day 55^64^, and Shih et al., using the same hiPSC line and NGN2 protocol, reported a peak in mean firing rate at days 30–36 followed by a decline until day 42^8^. The choice of hiPSC line could represent another important consideration. However, previous studies using NGN2 neurons from different hiPSC lines demonstrated comparable synaptic properties^34^ and similar clustering of MEA-derived activity parameters in PCA analyses^16^.

The results are further confined to the specific culture conditions used, including cell density and medium formulation. Alternative conditions may shift the optimal assay window or alter the relative performance of individual coatings. In addition, only coatings that are commonly used by the community were tested, whereas alternative substrates not included here, such as brain-derived decellularized matrices^29^, might provide more favorable conditions for long-term network stability and performance in these NGN2 neurons.

These constraints are likely to contribute to the apparent discrepancy between the present conclusions and those of earlier comparative studies. In our system, FN performed most consistently across functional readouts, yet LN and Matrigel also supported robust network formation, and the ranking was not uniform. When the astrocyte source was changed to donor-matched hiPSC-derived astrocytes, LN was less favorable than in co-culture with primary astrocytes.

Taken together, these observations argue against the existence of a single universally optimal coating. The substrate should instead be regarded as a model-specific parameter that requires empirical optimization for each cell type, culture format and readout. The present study establishes that FN is a suitable substrate for NGN2-induced neuron-astrocyte co-cultures on MEAs and, more generally, that FN merits inclusion in coating comparisons from which it has so far often been omitted.

Further, as with any extracellular recording technique, MEA measurements are susceptible to potential artefacts, including electrical noise, mechanical disturbances during medium exchange, and analysis-dependent effects related to spike detection and burst definition^65,66^. To minimize these influences as much as possible, the recordings in this study were conducted under stable environmental conditions, with medium changes and measurements carried out at specific intervals, and conservative spike detection parameters as recommended by the MEA manufacturer. Nonetheless, such factors should be considered when interpreting differences in activity patterns.

To complement MEA measurements and gain additional insights into the effects of different coatings on individual neuronal properties, techniques such as patch-clamp recording or calcium imaging can be used. While patch-clamp allows direct, high-temporal-resolution measurements of membrane and synaptic currents^67^, calcium imaging enables the simultaneous monitoring of activity in many individual neurons, thereby capturing both single-cell responses and network-level dynamics^68^. Moreover, combining MEA recordings with calcium imaging has been shown to provide complementary information^69^.

In summary, this study underlines that the coating is a pivotal, yet often overlooked, parameter in neuronal and neuron-astrocyte co-culture assays. Notably, FN, a rarely selected coating, supported robust electrical activity in hiPSC-derived NGN2 neurons, demonstrating that less-common coating may best suit specific applications. In addition, FN could be used as a human-derived alternative to Matrigel. The use of a single ECM protein instead of a complex ECM protein mixture may improve reproducibility and standardization in in vitro models. Coating selection should therefore be treated as a primary experimental parameter, particularly for drug screening platforms, as it can significantly alter cellular responses and thus the reliability of assay results. Optimizing the coating is thus not only a technical requirement, but also necessary for translating in vitro screening results into clinically relevant predictions.

## Methods

### Coatings

All coatings were diluted according to Table 1. For the coating procedure with the polymers PEI, PLO, PDL and PLL, culture plates were incubated overnight at 4 °C. The following day, plates were washed three times using H_2_O for PEI and PBS without calcium and magnesium (PBS–) for PLO, PDL, and PLL. The solutions were removed, and plates were air-dried under a laminar flow hood with open lids. The polymer-coated plates were used directly or further coated with LN. LN was incubated for 45 min at 37 °C. FN coated plates were incubated for one hour at room temperature. After incubation, the solution was aspirated, and plates were dried under laminar flow for two hours with open lids. For Matrigel coating, plates were incubated for 45 min at 37 °C. Prior to cell seeding, excess LN and Matrigel were aspirated.

**Table 1:** Overview of the tested coatings.

Coating	CAT#, source	Conc.	Dilution medium	Origin
PEI	P3143, Merck	0.1%	Borate buffer pH 8.4 (50 mM boric acid, 12.5 mM sodium tetraborate in dH_2_O)	synthetic
PLO	P4957, Merck	0.001%	PBS–	synthetic
PDL	P6407, Merck	0.1 mg/mL	dH_2_O	synthetic
PLL	P4707, Merck	0.001%	PBS--	synthetic
LN	L2020, Merck	1.5 µg/cm^2^	PBS--	murine
FN	FC010, Merck	20 µg/cm^2^	PBS--	human
Matrigel	CLS356231, Corning	12.5 µg/cm^2^	DMEM /F-12	murine

### hiPSC-derived NGN2 neurons

NGN2 neurons were differentiated from the hiPSC line BIONi010-C-13 (EBiSC; BioSample ID: SAMEA103988285, listed on hpscreg.eu) according to Wihan et al. 2023^70^. After two days of doxycycline induction for NGN2 overexpression, the cells were cryopreserved in CryoStor CS10 (STEMCELL Technologies) as single cells. For all experiments, three independent neuronal differentiation batches of NGN2 neurons were used as biological replicates. Cells were thawed and directly plated onto the different coatings (Table 1). Cells were seeded in a concentration of 0.1 × 10^6^ cells/cm^2^ in neurobasal medium (NBM) consisting of 50% DMEM/ F-12 + GlutaMAX™, 50% neurobasal medium, 0.5x serum-free supplement (50x), 0.5x serum-free supplement based on Bottenstein’s N-1 formulation (100x), 0.5x GlutaMAX™, 0.5x MEM non-essential amino acids solution (100x), 500 nM sodium pyruvate (100 mM), 50 nM 2-mercaptoethanol (50 mM), 0.025% human insulin solution, and 5 U/mL penicillin-streptomycin, supplemented with 10 µM Y-27,632 and 2 µg/mL doxycycline. The first three days after thawing, the medium was changed daily to NBM supplemented with 2 µg/mL doxycycline. On day four, the medium was changed to NBM+, consisting of NBM supplemented with 20 ng/mL brain derived neurotrophic factor (BDNF), 20 ng/mL glia cell derived neurotrophic factor (GDNF), 1 mM N6,2′-O-Dibutyryladenosine 3′,5′-cyclic monophosphate sodium salt and 200 µM L-ascorbic acid. Half media change was performed two times per week.

### Human primary astrocytes

Human primary astrocytes were purchased from ScienCell (#1800) and expanded according to manufactures instruction until passage 3. Astrocytes were cryopreserved in CryoStor CS10 (STEMCELL Technologies) and thawed directly prior to use. The plates were coated according to Table 1. The cells were seeded in a density of 0.1 × 10^6^ cells/cm^2^ in NBM+. The medium was changed two times per week.

### hiPSC-derived astrocytes

Astrocytes were differentiated from the hiPSC line BIONi010-C (EBiSC; BioSample ID: SAMEA3158050, listed on hpscreg.eu). Astrocyte progenitor cells were generated and further matured for two weeks according to Dittlau et al. with minor modifications^71^ and subsequently cryopreserved. Astrocytes were thawed in a 37 °C water bath and plated onto Matrigel-coated plates in Astrocyte Maturation Medium (AMM) composed of 1:1 of DMEM/F12, and neurobasal medium supplemented with 100 U/mL Pen/Strep, 1x N2, 1x NEAA, 0.8 µM L-Ascorbic acid, 2 mM L-Glutamine, 1 mM sodium pyruvate, and 2% FBS. The following growth factors were added freshly prior to media change: 200 ng/mL Insulin-like growth factor (IGF), 10 ng/mL Activin A and 10 ng/mL Heregulin β-1. Media change was performed every second day for 10 days, to further mature the astrocytes. On the day of co-culture initiation, astrocytes were dissociated using StemPro™ Accutase™ Cell Dissociation Reagent (Thermo Fisher Scientific, Gibco) and seeded on top of the neurons as described in the next session.

### Co-culture of NGN2 neurons and astrocytes

For ICC of the co-culture, neurons were seeded as previously described. On day four, astrocytes were seeded onto the neuronal culture in NBM + at a density of 0.1 × 10^6^ cells/cm^2^. For MEA experiments, electrodes were coated as described (15 µL per well). NGN2 neurons were seeded at a density of 30,000 cells per well in a 10 µL droplet of NBM supplemented with 10 µM Y-27,632. The plates were kept under a laminar flow hood for 10 min to allow cell attachment, followed by incubation at 37 °C for 30 min. Subsequently, 300 µL of NBM supplemented with 2 µg/mL doxycycline was added to each well of a 48-well CytoView MEA plate (Axion Biosystems). The medium was changed daily until day four. On day four, the medium was completely removed, and astrocytes were seeded at a density of 30,000 cells per well in a 10 µL droplet. The plate was again placed under a laminar flow for 10 min to allow cell sedimentation, followed by 30 min of incubation at 37 °C. Subsequently, 300 µL NBM + was added to each well. Half of the medium (NBM+) was changed two times per week.

### Image analysis of bright field images

Bright field images were acquired using the VAIDR microscope (TRI Thinking Research Instruments GmbH) on day 1, 4, 7, 10 and 14 after plating the cells. Images were consistently taken from approximately the same fields within each well to ensure comparability over time. For neurite outgrowth analysis, images were quantified using VAIDR’s built-in analysis software. The software was trained on example images to recognize and segment neurites, soma, and background regions automatically. From these segmentations, neurite and soma areas (in pixels per image) were obtained, and the ratio of neurite area to soma area was calculated to assess neurite development over time. Astrocyte confluency was analyzed using the identical microscope, timepoints and software. The software was trained to distinguish astrocyte-covered areas from the background. Based on this segmentation, the percentage of confluency was calculated.

### Fluorescein‑diacetate and propidium iodide staining

NGN2 neurons or astrocytes were washed once with PBS–. Subsequently the cells were stained with 0.5 µg/ml Fluorescein‑diacetate (FDA) and 0.5 µg/ml propidium iodide (PI) in PBS– for 10 s. The cells were washed once with PBS– after removing the FDA/PI solution and imaged directly with a Yokogawa CQ1 spinning disk confocal microscope. Fluorescent signals were quantified from a minimum of three images per condition per biological replicate. Quantification was performed with ImageJ (version 1.53t)^72^ using the ImageJ LDA_macro of Kerkhoff et al.^73^. FDA- and PI-positive cells were counted automatically, and the percentage of viable cells was calculated accordingly.

### Immunocytochemistry

Cells were washed once with PBS with calcium and magnesium (PBS++) and fixed using 4% paraformaldehyde (PFA). After two washing steps with PBS++, cells were incubated for 1 h in a permeabilization/blocking buffer containing 1% BSA and 0.2% TritonX in PBS++. Primary antibodies were diluted in the permeabilization/blocking buffer: mouse anti-beta III Tubulin 1:1000 (TUBB3, ab7751 abcam), rabbit anti-Ki67 1:500 (ab15580, abcam), mouse anti-MAP2 1:200 (13-1500, Thermo Fisher Scientific), mouse anti-GFAP 1:200 (MA5-12023, Thermo Fisher Scientific), rabbit anti-Tau 1:300 (ab76128, abcam). After incubation overnight at 4 °C, the cells were washed three times with blocking buffer containing 1% BSA in PBS++. All secondary antibodies were diluted 1:1000 in PBS++: goat anti-mouse Alexa Fluor 647 (A-21236, Thermo Fisher Scientific), goat anti-rabbit Alexa Fluor 488 (A-11034, Thermo Fisher Scientific), goat anti-mouse Alexa Fluor 488 (A-11001, Thermo Fisher Scientific), goat anti-rabbit Alexa Fluor 647 (A-21245, Thermo Fisher Scientific). After incubation for one hour at room temperature in the dark, the cells were washed two times with PBS + + and counterstained with 4′,6-diamidino-2-phenylindole DAPI (R37606, Thermo Fisher Scientific). Samples were imaged using a Yokogawa CQ1 spinning disk confocal microscope. Imaging settings such as laser power and exposure time were kept constant for each individual marker across all experimental conditions. Z-stacks were acquired and maximum intensity projections were generated for analysis and visualization.

### Image analysis of ICC images

Images were analyzed using ImageJ (version 1.53t). In general, brightness and contrast were adjusted uniformly across image sets (exemplary images shown in the figures). For quantitative analysis of Ki67-positive cells, DAPI- and Ki67-positive nuclei were counted using the particle analyzer. Images were thresholded and converted into binary masks; for DAPI, watershed segmentation was applied to separate adjacent nuclei, whereas for Ki67 the “fill holes” function was used prior to particle analysis.

### RT-qPCR

The cells were lysed in RLT buffer (Qiagen) and stored at -80 °C. Total RNA was extracted using the RNeasy Mini Kit (Qiagen) according to manufacturer’s instructions. RNA concentration and purity were determined using a NanoDrop spectrophotometer (Thermo Fisher Scientific). For cDNA synthesis, 40 to 400 ng of total RNA was reverse transcribed using the High-Capacity cDNA Reverse Transcription Kit (10400745, Thermo Fisher Scientific) following the manufacturer’s protocol. The resulting cDNA was diluted in nuclease-free water to a concentration of 2 µg/µL and stored at − 20 °C until further use. Quantitative Real-Time PCR (qPCR) was performed using the TaqMan Advanced Master-Mix (13418456, Thermo Fisher Scientific) and gene-specific TaqMan assays (see Table 2) according to the manufacturer’s instructions. Reactions were carried out in 96-well plates using 2 µg of DNA per reaction, in a final volume of 10 µL, on the QuantStudio 7 Flex (Thermo Fisher Scientific). Each reaction was performed in technical duplicates. Relative gene expression was calculated using the ΔΔCt method, with the housekeeping genes as endogenous control (GAPDH, HPRT, GUSB for neuronal cells; HPRT, GUSB for astrocytes).

**Table 2:** Information on used TaqMan assays from Thermo Fisher Scientific for qPCR.

Gene	TaqMan Assay ID
*GFAP*	Hs00909233_m1
*NES*	Hs04187831_g1
*MAP2*	Hs00258900_m1
*DLG4*	Hs01555373_m1
*SYN1*	Hs00199577_m1
*MAPT*	Hs00902194_m1
*HPRT*	Hs99999909_m1
*GUSB*	Hs99999908_m1
*GAPDH*	Hs99999905_m1

### MEA recording and data analyses

MEA recordings were performed weekly using the Maestro Pro system (Axion Biosystems) at 37 °C and 5% CO₂. Spontaneous neural activity was recorded for five minutes using Axion’s MEA software. Data were sampled at 12.5 kHz and analyzed using the AxIS Software (Axion Biosystems). A high-pass filter at 200 Hz and a low-pass filter at 3 kHz were applied. Active electrodes were defined as electrodes with a minimum spike rate of 5 spikes/min. Spike detection was performed using the Adaptive Threshold Crossing method. The threshold was set to six times the standard deviation of the noise. Spike counts were computed in 1-second intervals (binning). Burst detection was performed using the Poisson Surprise algorithm with a minimum surprise value of 5. Network bursts were identified with a maximum inter-spike interval of 100 ms, requiring a minimum of 10 spikes and at least 25% of electrodes participating. A synchrony window of 20 ms was applied. Data was exported as csv file and further processed and visualized using OriginPro 2024b (OriginLab Corporation, Northampton, MA, USA). The Mean Firing Rate (Hz) was calculated as the total number of spikes divided by the total number of electrodes (16) and the recording duration (5 min). Network Inter-Burst Interval (IBI) coefficient of variation (CoV) was calculated by the standard deviation divided by the average of the time between network bursts. This parameter defines network burst regularity. Smaller values indicate higher regularity. The Synchrony Index was determined using AxIS Software (Axion Biosystems) based on the method described by Paiva et al. (2010)^42^. This binless measure quantifies the temporal alignment of spike trains across electrodes, with values ranging from 0 (no synchrony) to 1 (perfect synchrony). A synchrony window of 20 ms was used, as specified in the analysis settings.

For selected MEA parameters, the CoV was calculated using OriginPro 2024b (OriginLab Corporation, Northampton, MA, USA). CoV was defined as the ratio of the standard deviation to the mean (SD/mean) and used to assess variability across the wells per condition.

Principal component analysis (PCA) was performed in OriginPro 2024b (OriginLab Corporation, Northampton, MA, USA). Eight MEA parameters were included in the analysis (mean firing rate, number of bursts, burst duration, burst percentage, number of network bursts, synchrony index, inter-burst interval, and network IBI CoV). Data was centered and standardized (z-score transformation), and the analysis was based on the correlation matrix. PCA was carried out separately for week 3 and week 6 data sets. Results were visualized using PCA biplots.

### Statistical analysis

Statistical analysis and data visualization was performed with OriginPro 2024b (OriginLab Corporation, Northampton, MA, USA). Due to the small sample size (*n* = 3), no formal test for normality was conducted. A normal distribution of the data was assumed based on the overall distribution characteristics. A parametric test (one-way ANOVA with Tukey’s multiple comparison) was used unless stated otherwise in the figure legends. Statistical significance is indicated by: *p < 0.05, **p < 0.01, ***p < 0.001. Data are presented as mean ± SD if not stated otherwise in the figure legends.

## Supplementary Information

Below is the link to the electronic supplementary material.

Supplementary Material 1

## Data Availability

All data generated during the current study is available from the corresponding author on reasonable request.
