# Supplementary material for: Comparative analysis of surface coatings for human neural co-cultures identifies fibronectin as a robust MEA substrate
Source: Sci Rep. 2026 Aug 20;16:26201. doi: 10.1038/s41598-026-67677-6 (PMC13493967; doi:10.1038/s41598-026-67677-6)
Supplement: Supplementary file 1 [file 41598_2026_67677_MOESM1_ESM.docx]

**Comparative Analysis of Surface Coatings for Human Neural Co-Cultures identifies Fibronectin as a Robust MEA Substrate**

Isabell Karnatz^1,2,3^, Ida Keller Skousen^4^, Benjamin Schmid^4^, Christian Clausen^4^, Bjørn Holst^4^ Heiko Zimmermann^1,2,3^*, Jeanette Wihan^1,2†^, Julia C. Neubauer^1,2†^

^1^ Fraunhofer Institute for Biomedical Engineering IBMT, 66280 Sulzbach, Germany;

isabell.karnatz@ibmt.fraunhofer.de (I.K.); heiko.zimmermann@ibmt.fraunhofer.de (H.Z.); jeanette.wihan@ibmt.fraunhofer.de (J.W.); julia.neubauer@ibmt.fraunhofer.de (J.C.N.)

^2^ Fraunhofer Project Center for Stem Cell Process Engineering, 97070 Wuerzburg,

^3^ Molecular and Cellular Biotechnology/Nanotechnology, Saarland University, 66123 Saarbruecken, Germany

^4^ Bioneer A/S, Kogle Allé 2, 2970 Hørsholm, Denmark; [iks@bioneer.dk](mailto:iks@bioneer.dk) (I.K.S.); bsc@bioneer.dk (B.S.); ccl@bioneer.dk (C.C.)

* corresponding author

^†^ authors contributed equally

**Supplementary Figures**

**Neurons express key neuronal markers in both protein and gene expression analyses**

**Supplementary Figure 1: Maturation of hiPSC-derived neurons on selected coatings over 14 days.** **a** Representative immunocytochemistry images of neuronal cells on day 14, stained for DAPI, TUBB3 and MAP2. Neurons show a similar TUBB3 and MAP2 marker expression across all conditions. **b** Gene expression analysis via RT-qPCR on day 14 for general neuronal (*MAP2, MAPT*), pre-synaptic (*SYN1*) and post-synaptic (*DLG4*) marker. Fold change of the gene expression compared to neurons on day 0 is shown (n = 3). Data are presented as mean ± SEM. Statistical analysis: One-way ANOVA followed by Tukey’s post hoc test (p ≤ 0.05). ns, not significant; PEI, Polyethylenimine; PLO, Poly-L-Ornithine; PDL, Poly-D-Lysine; PLL, Poly-L-Lysine; LN, Laminin; FN, Fibronectin

**Supplementary Figure 2: Representative spike activity raster plots of MEA recordings from selected wells at week 3 and week 6.** Active electrodes were defined as electrodes with a minimum spike rate of 5 spikes/min. Each tick mark represents a detected spike on an individual electrode. Spikes were detected using adaptive threshold crossing (threshold = 6× SD of noise). Bursts, highlighted in blue, were identified using the Poisson Surprise algorithm (minimum surprise value = 5) and applied consistently across all recordings. Magenta boxes indicate network bursts, defined by a maximum inter-spike interval of 100 ms, a minimum of 10 spikes, and at least 25% electrode participation. PEI, Polyethylenimine; PLO, Poly-L-Ornithine; PDL, Poly-D-Lysine; PLL, Poly-L-Lysine; LN, Laminin; FN, Fibronectin

**Supplementary Figure 3: Representative extracellular spike waveforms across coating conditions and time points.** Waveforms from co-cultures cultivated on different substrate coatings are shown at week 1, week 3, and week 6. Pre-processed spike files (.spk) exported from Axion Navigator were converted to .nex format and visualized using Plexon Offline Sorter. For each coating condition, a representative electrode was selected and displayed consistently across all timepoints, with all spike waveforms detected during the recording period (300 s) overlaid. PEI, Polyethylenimine; PLO, Poly-L-Ornithine; PDL, Poly-D-Lysine; PLL, Poly-L-Lysine; LN, Laminin; FN, Fibronectin

**Supplementary Figure 4: Batch-to-batch variability of MEA recordings.** Data of three independent neuronal differentiation batches are shown for different MEA parameters at week 3 and week 6 of cultivation. Data are presented as mean ± SD (N = 4 for all conditions except PLO-LN, where N = 3). Statistical analysis: One-way ANOVA followed by Tukey’s post hoc test (p ≤ 0.05). PEI, Polyethylenimine; PLO, Poly-L-Ornithine; PDL, Poly-D-Lysine; PLL, Poly-L-Lysine; LN, Laminin; FN, Fibronectin

| Study | Cell source (species) | Neuronal subtype | Coatings tested | Best-performing coating | Outcome measures | Key conclusion |
| --- | --- | --- | --- | --- | --- | --- |
| Qian *et al.* 2004 [1] | MSC (human) | Neuron-like | PDL, PLL, collagen, LN, FN, Matrigel | Matrigel | ICC, bright-field microscopy, expansion efficacy | Complex ECM mixtures favor adhesion and expansion |
| Flanagan *et al.* 2006 [2] | NSPC (human, mouse) | n.r. | PLO, FN, LN, Matrigel | LN | Migration, expansion, differentiation, neurite length | LN and its α6/β1 integrin receptors regulate human NSPCs |
| Ma *et al.* 2008 [3] | hESC | Neural progenitors and neurons (n.r.) | PDL, PDL-LN, PDL-FN, collagen I, Matrigel | PDL-LN (and LN-rich Matrigel) | Neurite outgrowth, progenitor expansion | LN acts via α6β1 integrin; FN inferior for progenitor expansion |
| Sun *et al*. 2012 [4] | Primary (rat) | Hippocampal | Matrigel, LN, FN, PEI, PDL, PLL | No universal best | Neurite outgrowth, ICC | Coating is a key determinant of network structure; optimum depends on endpoint |
| Tonge *et al.* 2012 [5] | Primary (mouse) | Cortical and hippocampal | FN, LN, merosin | FN | Neurite outgrowth, axonal regeneration, integrin blockade | FN supports outgrowth of adult CNS neurons via α5β1 integrin |
| Muratore *et al.* 2014 [6] | hiPSC | Cortical (dual SMAD) | PLO-LN, Matrigel | Matrigel | ICC, RT-qPCR | Matrigel favors cortical differentiation |
| Setien *et al.* 2020 [7] | iPSC (rat) | n.r. | PDL, PLO-LN, gelatin, Geltrex | PLO-LN | Patch clamp, ICC, neurite outgrowth | PLO-LN supports electrophysiological maturation |
| Li et al. 2024 [8] | hiPSC | Cortical (NGN2-induced) | PDL, PLO, LN, Matrigel and double combinations | PDL-Matrigel | Neurite outgrowth, ICC | Double coating reduces cell clumping and improves purity; single LN/Matrigel causes clumping |
| Yang *et al.* 2024 [9] | hiPSC | Motor | PLO-LN, PLO-LN-FN, PLO-LN-FN-Matrigel, PLO-Matrigel, PEI | PLO-Matrigel (maturation speed); PEI (uniformity) | MEA | Coating determines both maturation rate and variability of electrophysiological signals |
| Wu *et al.* 2024 [10] | NSC (mouse) | n.r. | PLL, Matrigel | Matrigel | Adhesion, migration, proliferation assays | Matrigel favors adhesion and migration |
| Grasselli *et al.* 2025 [11] | hiPSC | Cortical (NGN2-induced) | Chitosan, Matrigel | Chitosan / Matrigel | ICC, western blot, MEA (only Chitosan) | Chitosan is a viable alternative to PLO-LN for functional network development |
| Present study | hiPSC | Cortical (NGN2-induced) | PEI, PLO, PDL, PLL, each ± LN, LN, FN, Matrigel | LN, FN, Matrigel for structure; FN for functional robustness | Neurite outgrowth, viability, Ki-67, RT-qPCR, ICC, MEA over 6 weeks, PCA, CoV | ECM-only coatings outperform polymer-only; LN, FN and Matrigel all supported robust networks, with FN showing the lowest variability and highest active-electrode retention |

**Supplementary Table 1:** **Overview of studies comparing surface coatings for neural cell culture.** Studies are ordered by publication year. The present study is marked in grey. All studies used CNS neural cells. ICC, immunocytochemistry; MEA, micro-electrode array; NSPC, neural stem/precursor cell; n.r., not reported.

**References**

1.	Qian, L. & Saltzman, W. M. Improving the expansion and neuronal differentiation of mesenchymal stem cells through culture surface modification. *Biomaterials* **25**, 1331–1337 (2004).

2.	Flanagan, L. A., Rebaza, L. M., Derzic, S., Schwartz, P. H. & Monuki, E. S. Regulation of human neural precursor cells by laminin and integrins. *J. Neurosci. Res.* **83**, 845–856 (2006).

3.	Ma, W. *et al.* Cell-extracellular matrix interactions regulate neural differentiation of human embryonic stem cells. *BMC Dev. Biol.* **22**, 90 (2008).

4.	Sun, Y. *et al.* Surface coating as a key parameter in engineering neuronal network structures in vitro. *Biointerphases* **7**, 1–14 (2012).

5.	Tonge, D. *et al.* Fibronectin supports neurite outgrowth and axonal regeneration of adult brain neurons in vitro. *Brain Res.* **1453**, 8–16 (2012).

6.	Muratore, C. R., Srikanth, P., Callahan, D. G. & Young-Pearse, T. L. Comparison and Optimization of hiPSC Forebrain Cortical Differentiation Protocols. *PLoS One* **9**, (2014).

7.	Setien, M. B. *et al.* Differentiation and characterization of neurons derived from rat iPSCs. *J. Neurosci. Methods* **338**, (2020).

8.	Li, S., Liu, Y., Luo, X. & Hong, W. Systematic Evaluation of Extracellular Coating Matrix on the Differentiation of Human-Induced Pluripotent Stem Cells to Cortical Neurons. *Int. J. Mol. Sci.* **26**, (2024).

9.	Yang, M. *et al.* Polyethyleneimine facilitates the growth and electrophysiological characterization of iPSC-derived motor neurons. *Sci. Rep.* **14**, 26106 (2024).

10.	Wu, C. *et al.* Cellular and Molecular Insights into the Divergence of Neural Stem Cells on Matrigel and Poly-l-lysine Interfaces. *ACS Appl. Mater. Interfaces* **16**, 31922–31935 (2024).

11.	Grasselli, S., Di Lisa, D., Ferrari, P. F., Martinoia, S. & Pastorino, L. Chitosan suitability to adhesion, neuronal differentiation and maturation of hiPSCs compared to Matrigel coated substrates. *Carbohydr. Polym. Technol. Appl.* **9**, 100686 (2025).
